# Effectiveness and Safety of Contemporary Drug-Eluting Stents in Patients With Diabetes Mellitus

**DOI:** 10.1016/j.jacasi.2021.07.009

**Published:** 2021-09-21

**Authors:** Yujin Yang, Junho Hyun, Junghoon Lee, Ju Hyeon Kim, Jeong Bok Lee, Do-Yoon Kang, Pil Hyung Lee, Jung-Min Ahn, Duk-Woo Park, Seung-Jung Park, IRIS-DES Registry Investigators

**Affiliations:** a Department of Cardiology, Asan Medical Center, University of Ulsan College of Medicine, Seoul, South Korea; b Division of Clinical Epidemiology and Biostatistics, Center for Medical Research and Information, Asan Medical Center, University of Ulsan College of Medicine, Seoul, South Korea

**Keywords:** coronary artery disease, diabetes mellitus, drug-eluting stent, percutaneous coronary intervention, DES, drug-eluting stent(s), DM, diabetes mellitus, HbA_1c_, glycosylated hemoglobin, MACE, major adverse cardiovascular event(s), MI, myocardial infarction, PCI, percutaneous coronary intervention, PES, paclitaxel-eluting stent(s), SES, sirolimus-eluting stent(s), TVF, target vessel failure, TVR, target vessel revascularization

## Abstract

**Background:**

Diabetes mellitus (DM) is a well-known risk factor for adverse cardiovascular events in patients receiving percutaneous coronary intervention (PCI). Limited data are available on the relative performance of different types of contemporary drug-eluting stents (DES) for diabetic patients.

**Objectives:**

The authors investigated the effectiveness and safety profiles of several contemporary DES in patients with DM in a “real-world” clinical setting.

**Methods:**

Among 24,516 patients enrolled in a multicenter, prospective registry, 7,823 patients with DM were treated with 4 contemporary DES: 2,877 with a cobalt chromium everolimus-eluting stent (EES), 789 with a biodegradable polymer biolimus-eluting stent, 2,286 with a platinum chromium-EES, and 1,871 with a Resolute zotarolimus-eluting stent. The primary outcome was target vessel failure (TVF) (a composite of cardiac death, target vessel myocardial infarction, and target vessel revascularization).

**Results:**

The median follow-up duration was 2.9 years. Observed 3-year rates of TVF were not significantly different according to different DES types. On multigroup propensity-score analysis, the adjusted HRs for TVF were similar in between-group comparisons: biodegradable polymer biolimus-eluting stent (HR: 0.94; 95% CI: 0.76-1.16; *P* = 0.57), platinum chromium-EES (HR: 0.94; 95% CI: 0.81-1.09; *P* = 0.41), and Resolute zotarolimus-eluting stent (HR: 1.01; 95% CI: 0.86-1.18; *P* = 0.93) compared with the cobalt chromium-EES (reference). This trend was maintained in patients with non–insulin- and insulin-treated DM.

**Conclusions:**

In this multicenter clinical-practice PCI registry, no significant between-group differences were found for a 3-year risk of TVF in patients with DM undergoing PCI with various types of contemporary DES. (Evaluation of the First, Second, and New Drug-Eluting Stents in Routine Clinical Practice [IRIS-DES]; NCT01186133)

Diabetes mellitus (DM) remains a clinically relevant risk factor for short- and long-term adverse events after percutaneous coronary intervention (PCI). Hyperglycemia and insulin resistance in DM trigger platelet dysfunction and a proinflammatory response, thus accelerating atherosclerosis and causing adverse coronary events such as repeat revascularization and myocardial infarction (MI) in patients with DM. For both diabetic and non-diabetic patients, drug-eluting stents (DES), as compared with bare-metal stents, show better clinical outcomes with reduced risks of mortality, MI, and repeat revascularization (1).

In recent decades, DES have evolved rapidly, and previous limitations have been overcome by modifying polymers, strut design, and size; thus, newer DES became thinner, delivering drugs more efficiently and triggering less inflammation (2). Real-world data showed that second-generation DES provided better clinical outcomes with reduction of MI, repeat revascularization, and stent thrombosis, compared with early-generation DES (3). However, DM is still a significant determinant of poor clinical outcomes in contemporary PCI practice. Moreover, the superiority of newer-generation DES is not always evident in patients with DM (4). Until recently, limited data were available on the relative performance between the different types of contemporary DES in high-risk patients with DM. We, therefore, compared the effectiveness and safety profiles of several contemporary DES in patients with DM, using data from a large-sized, “real-world” clinical-practice PCI registry.

## Methods

### Study population

The study population was derived from the IRIS-DES (Interventional Cardiology Research In-Cooperation Society-Drug-Eluting Stents) registry (NCT01186133). The study design of the IRIS-DES registry and the related consecutive analyses have been described previously (5, 6, 7, 8, 9). Briefly, the IRIS-DES study involved prospective, stent-specific, multicenter recruitment of unrestricted patients undergoing PCI with DES in Korea and consisted of several arms of first- and second-generation DES in a real-world setting. In this registry, the exclusion criteria were minimal. They are listed as follows: 1) history of cardiogenic shock; 2) diagnosed with malignancy or other comorbid conditions and had a life expectancy <12 months; 3) treatment with a combination of different DES types; 4) presented with active bleeding, thus contraindicating treatment with dual-antiplatelet therapy; and 5) had undergone or were scheduled to undergo planned surgery, therefore necessitating interruption of antiplatelet therapy within 6 months after PCI.

The current analysis included patients treated using 4 different types of second-generation, contemporary DES: a cobalt chromium everolimus-eluting stent (CoCr-EES; Xience V, Prime, Xpedition, or Alpine model, Abbott Vascular), a biodegradable polymer biolimus-eluting stent (BP-BES; BioMatrix, Biosensors; or Nobori, Terumo Clinical Supply), a platinum chromium-EES (PtCr-EES; Promus Element, Premier, or Synergy model, Boston Scientific), and a Resolute zotarolimus-eluting stent (Re-ZES; Resolute Integrity, Endeavor, or Onyx model, Medtronic). The registries were supported by the CardioVascular Research Foundation, Seoul, Korea, and there were no profit-involved interests in the design, conduct, or analysis of this study. The ethics committee of each participating center approved the study protocol (IRB no. 2014-0154), and all patients provided written informed consent.

### PCI procedures and clinical follow-up

In each stent-specific registry, PCI was performed according to standard techniques at the discretion of each operator. The registry did not specify stent types according to clinical or anatomical characteristics; therefore, the choice of specific DES type was at the discretion of each operator. However, the study protocol specified that the type of stent implanted into the target lesion was also to be implanted into other non-target lesions. Periprocedural anticoagulant agents were administered according to standard regimens. Administration of glycoprotein IIb/IIIa inhibitors was at the treating physician’s discretion. All patients undergoing PCI received a loading dose of aspirin and P2Y_12_ receptor inhibitor (clopidogrel, prasugrel, or ticagrelor) before or during the PCI procedure. After the intervention, aspirin was continued indefinitely, and P2Y_12_ receptor inhibitors were prescribed for at least 12 months regardless of the DES type. During follow-up, guideline-directed medical therapy and management of risk factors for secondary prevention were highly recommended for all patients. All patients with DM were treated by adopting the newest clinical practice guidelines and managed by endocrinologists to maintain adequate glycemic control.

Clinical follow-up was done during hospitalization and at 30 days, 6 and 12 months, and every 6 months after that. At each visit, data of patients’ clinical status, interventions, and outcomes were recorded. All baseline characteristics and outcome data were kept in a dedicated, electronic case report form by specialized personnel at each participating center. Thus, the internet-based system provided each center with immediate and continuous feedback on the processes and quality of care measurements. Registry data were periodically monitored and verified in the participating hospitals by the academic coordinating center members (Clinical Research Center, Asan Medical Center, Seoul, Korea) (5,9).

### Study outcomes and definitions

The primary outcome of the study was target vessel failure (TVF), which was defined as a composite of cardiac death, target vessel MI, and clinically driven target vessel revascularization (TVR). Secondary outcomes included individual components of the primary outcome: death from any cause; any MI; any revascularization; stent thrombosis; and major adverse cardiac event (MACE; a composite of all-cause mortality, any MI, or any revascularization).

The cause of death was considered to be cardiac-related unless a definite noncardiac cause could be established. Periprocedural MI was defined by an elevation of the creatine kinase-myocardial band fraction to more than 5 times the upper normal limit within the first 48 hours of the index revascularization procedure with ischemic symptoms and signs. As per definition, periprocedural MI is included in target vessel MI and any MI. Repeat revascularization included any type of percutaneous or surgical revascularization procedure and was categorized as TVR or non-TVR. Stent thrombosis (definite or probable) was defined as a thrombotic occlusion of a coronary stent according to the Academic Research Consortium definition. All outcomes of interest were confirmed by source documentation collected at each hospital and were centrally adjudicated by an independent clinical events committee whose members were blinded to the study devices.

### Statistical analysis

Baseline characteristics, including patient demographics, comorbidities and risk factors, cardiac status, clinical presentation, and anatomical and procedural features are described according to different types of DES. Categorical variables are presented as numbers (percentages), with differences among treatment groups analyzed by the chi-square or Fisher exact test, as appropriate. Continuous variables are presented as mean ± SD, with differences among treatment groups evaluated by analysis of variance.

Cumulative events of clinical outcomes were assessed using the Kaplan-Meier method and compared using the log-rank test. All analyses were truncated at 3 years of follow-up owing to the different follow-up durations according to DES type and the small number of patients with data thereafter. A propensity-score weighting method was applied to control imbalances in different baseline characteristics across the different stent groups to minimize the confounding effect and selection bias in estimating the treatment effect in observational data. In this study, multiple treatment propensity scores were applied using the Toolkit for Weighting and Analysis of Nonequivalent Groups (TWANG) method (9,10). The corresponding inverse probabilities of treatment weight (the reciprocals of the propensity scores) were estimated by using generalized boosted models through an iterative estimation procedure (n = 3,000), using 10 related baseline characteristics: age, sex, previous MI, chronic kidney disease, ejection fraction <40%, clinical presentation as acute coronary syndrome, multi-vessel disease, total number of stent, and use of intravascular ultrasound, medication (statin, aspirin, P2Y_12_ inhibitor). The PROC SURVEYPHREG procedure of SAS software version 9.4 (SAS Institute) was used to correctly interpret weights as probability weights to evaluate the relative treatment effects. Lastly, we performed a subgroup analysis to evaluate the clinical outcomes according to the degree of DM control in patients with valid information on glycosylated hemoglobin (HbA_1c_). All reported *P* values were 2-sided and had not been adjusted for multiple testing. Owing to the potential for a type I error due to multiple comparisons, all findings in this study should be interpreted as exploratory. All analyses were performed using SAS software version 9.4 and R software version 3.2.2 13 (R Foundation for Statistical Computing).

## Results

### Baseline characteristics of the study population

**Figure 1:**
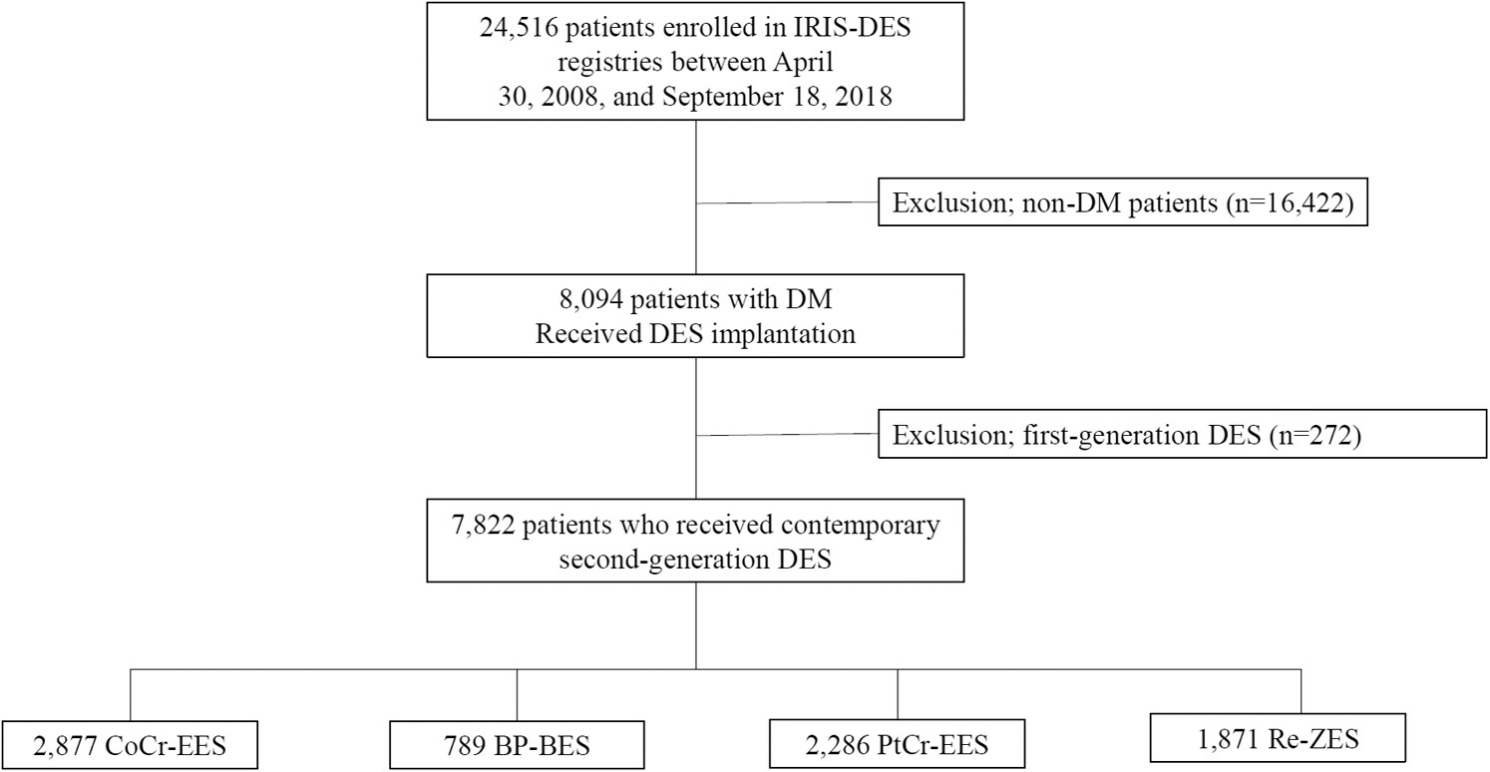
Flow Diagram BP-BES = biodegradable polymer biolimus-eluting stents; CoCr-EES = cobalt chromium everolimus-eluting stents; DES = drug-eluting stents; DM = diabetes mellitus; IRIS-DES = Interventional Cardiology Research Incorporation Society-Drug-Eluting Stents registry; PtCr-EES = platinum chromium everolimus-eluting stents; Re-ZES = Resolute zotarolimus-eluting stents.

A flow diagram of the study is shown in Figure 1. Among 24,516 patients enrolled in the IRIS-DES registry between April 30, 2008, and September 18, 2018, a total of 8,094 (33.0%) patients had DM. Among them, a total of 7,823 patients who received PCI with contemporary second-generation DES were finally included in the current study (2,877 with CoCr-EES, 789 with BP-BES, 2,286 with PtCr-EES, and 1,871 with Re-ZES). Among 7,823 patients with DM, 893 (11.4%) were treated with insulin therapy.

Baseline demographics, and clinical, anatomical, and procedural characteristics of the study population according to the different types of DES are shown in Table 1. Overall, there were significant between-group differences in age, sex, key clinical factors (dyslipidemia, current smoking, family history of coronary artery disease, prior history of MI or PCI, clinical presentation), and several anatomical and procedural characteristics. For medication data, aspirin and P2Y_12_ inhibitors were prescribed in over 98% of patients, and there was no difference among stent groups. However, the prescription rate of statin was about 90% of patients and tended to be lower in the BP-BES group. This pattern was also similar in 6,930 patients with non–insulin-treated DM (Supplemental Table 1) and 893 patients with insulin-treated DM (Supplemental Table 2). After multiple-treatment propensity-score weighting with the TWANG method, the balance of the pretreatment covariates was assessed, and significant improvement in baseline covariates was achieved after weighting (Supplemental Figure 1).

**Table 1:** Baseline Clinical, Angiographic, and Procedural Characteristics According to Different Stent Type

	Patients With Diabetes (N = 7,883)				
	CoCr-EES (n = 2,877)	BP-BES (n = 789)	PtCr-EES (n = 2,286)	Re-ZES (n = 1,871)	*P* Value
Age, y	65.4 ± 9.7	66.1 ± 10.1	66.2 ± 9.9	65.9 ± 9.9	0.03
Male	1,941 (67.5)	500 (63.4)	1,495 (65.4)	1,272 (68.0)	0.05
Body mass index, kg/m^2^	24.9 ± 3.3	24.8 ± 3.2	24.8 ± 3.3	24.9 ± 3.3	0.58
Hypertension	2,159 (75.0)	579 (73.4)	1,692 (74.0)	1,440 (77.0)	0.11
Dyslipidemia	1,587 (55.2)	277 (35.1)	1,329 (58.1)	1,157 (61.8)	<0.001
Current smoking	763 (26.5)	195 (24.7)	514 (22.5)	439 (23.5)	0.006
Family history of CAD	144 (5.0)	29 (3.7)	100 (4.4)	111 (5.9)	0.04
Previous MI	173 (6.0)	38 (4.8)	173 (7.6)	122 (6.5)	0.03
Previous heart failure	83 (2.9)	20 (2.5)	82 (3.6)	64 (3.4)	0.33
Previous PCI	507 (17.6)	79 (10.0)	444 (19.4)	338 (18.1)	<0.001
Previous CABG	74 (2.6)	18 (2.3)	40 (1.8)	45 (2.4)	0.25
Chronic kidney disease	250 (8.7)	48 (6.1)	186 (8.1)	159 (8.5)	0.12
Previous cerebrovascular disease	256 (8.9)	74 (9.4)	197 (8.6)	158 (8.4)	0.86
Peripheral vascular disease	81 (2.8)	20 (2.5)	74 (3.2)	67 (3.6)	0.36
Chronic lung disease	59 (2.1)	22 (2.8)	44 (1.9)	44 (2.4)	0.46
Ejection fraction, %	57.76 ± 11.32	57.86 ± 11.18	57.69 ± 10.89	57.58 ± 11.20	0.93
Clinical presentation					<0.001
Stable angina	1,440 (50.1)	345 (43.7)	935 (40.9)	841 (45.0)	
Unstable angina	803 (28.9)	237 (30.0)	778 (34.0)	604 (32.3)	
NSTEMI	355 (12.3)	116 (14.7)	355 (15.5)	273 (14.6)	
STEMI	279 (9.7)	91 (11.5)	218 (9.5)	153 (8.2)	
Number of diseased vessels	1.9 ± 0.8	1.7 ± 0.8	1.8 ± 0.8	1.9 ± 0.8	<0.001
Multivessel disease	1,757 (61.1)	412 (52.2)	1,323 (57.9)	1,139 (60.9)	<0.001
Moderate to severe calcification	338 (11.8)	55 (7.0)	287 (12.6)	196 (10.5)	<0.001
Complex lesion, ACC/AHA B2 or C type	2,307 (80.2)	554 (70.2)	1,680 (73.5)	1,504 (80.4)	<0.001
Bifurcation lesion	1,013 (35.2)	246 (31.2)	519 (22.7)	613 (32.8)	<0.001
De novo lesion	2,717 (94.4)	775 (98.2)	2,187 (95.7)	1,781 (95.2)	<0.001
Diffuse lesion	1,727 (60.0)	307 (38.9)	1,185 (51.8 )	1,077 (57.6)	<0.001
Number of stents per patient	1.8 ± 1.0	1.5 ± 0.8	1.6 ± 0.9	1.7 ± 0.9	<0.001
Total stent length per patient	34.1 ± 19.4	25.6 ± 11.9	30.3 ± 15.8	32.3 ± 17.0	<0.001
Average stent diameter	3.2 ± 0.4	3.1 ± 0.4	3.1 ± 0.4	3.1 ± 0.4	<0.001
IVUS	1,405 (48.8)	231 (29.3)	769 (33.6)	879 (47.0)	<0.001
Femoral approach	1,793 (62.3)	446 (56.5)	1,115 (48.8)	997 (53.8)	<0.001
Medication data					
Statin	2,518 (87.5)	669 (84.8)	2,069 (90.5)	1,712 (91.5)	<0.001
Aspirin	2,837 (98.6)	775 (98.2)	2,246 (98.3)	1,847 (98.7)	0.53
P2Y_12_ inhibitors	2,831 (98.4)	771 (97.7)	2,252 (98.5)	1,847 (98.7)	0.29
HbA_1c_ at admission	7.5 ± 1.5	7.5 ± 1.5	7.5 ± 1.5	7.4 ± 1.4	0.25

Values are mean ± SD or n (%).

ACC/AHA = American College of Cardiology/American Heart Association; BP-BES = biodegradable polymer biolimus-eluting stent; CABG = coronary artery bypass graft; CAD = coronary artery disease; CoCr-EES = cobalt chromium everolimus-eluting stent; HbA_1c_ = glycosylated hemoglobin; IVUS = intravascular ultrasound; MI = myocardial infarction; NSTEMI = non–ST-segment elevation myocardial infarction; PCI = percutaneous coronary intervention; PtCr-EES = platinum chromium everolimus-eluting stent (Synergy stent); Re-ZES = Resolute zotarolimus-eluting stent; STEMI = ST-segment elevation myocardial infarction.

### Clinical outcomes

The median duration of clinical follow-up was 2.9 years (interquartile range: 1.0 to 4.9 years). During the follow-up, there were 451 deaths (8.0%; 291 cardiac deaths [5.3%] and 160 non-cardiac deaths [2.7%]), 496 MIs (6.7%; 467 target vessel MIs [6.3%] and 29 non–target vessel MI [0.4%]), 561 repeat revascularizations (10.0%; 342 TVR [6.1%] and 219 non-TVR [3.9%]), and 24 stent thrombosis (0.4%). Overall, 993 patients (15.5%) experienced at least 1 TVF event, and 1,331 patients (21.3%) experienced at least 1 MACE.

**Figure 2:**
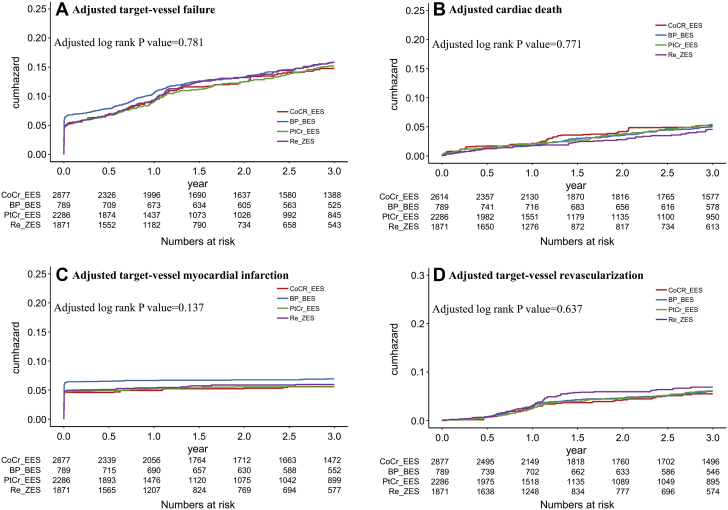
Unadjusted Curves for the TVF and its Components Crude cumulative-incidence curves for primary composite outcome of target vessel failure (TVF) **(A)** and its individual components: cardiac death **(B)**, target vessel myocardial infarction **(C)**, and target vessel revascularization **(D)**. *P* values were calculated using the log-rank test. Target vessel failure was defined as death from cardiac causes, target vessel myocardial infarction (MI), or target vessel revascularization. Abbreviations as in Figure 1.

Unadjusted and adjusted analyses of the primary composite outcome of TVF and its components at 3 years are shown in Table 2. The 3-year rate of TVF did not differ significantly among the different types of DES (16.3% for CoCr-EES, 14.4% for BP-BES, 14.9% for PtCr-EES, and 15.8% for Re-ZES; log-rank *P* value = 0.37) (Figure 2). The cumulative 3-year rates of cardiac death or TVR were similar among the groups. However, the rates of target vessel MI were different among the groups (lowest for BP-BES [4.7%] and highest for CoCr-EES [7.2%]; *P* = 0.005), mostly driven by periprocedural MI; the incidence of target vessel MI, except for periprocedural MI, was not different among the groups.

**Table 2:** Unadjusted and Adjusted Probabilities of the Primary Endpoint and its Components

Outcomes	Number of Events	3-y Event Rate^a^ (% and 95% CI)	Unadjusted		Multigroup Propensity-Score Analysis	
			HR (95% CI)	*P* Value	HR (95% CI)	*P* Value
Target vessel failure^b^						
CoCr-EES	404	16.3 (14.9-17.9)	1.00 (Referent)		1.00 (Referent)	
BP-BES	106	14.4 (12.0-17.1)	0.89 (0.73-1.09)	0.25	0.96 (0.78-1.19)	0.72
PtCr-EES	263	14.9 (13.3-16.8)	0.89 (0.77-1.03)	0.13	0.94 (0.81–1.10)	0.43
Re-ZES	220	15.8 (13.8-18.0)	0.99 (0.84-1.15)	0.85	1.02 (0.87-1.20)	0.79
Cardiac death						
CoCr-EES	110	5.2 (4.3-6.2)	1.00 (Referent)		1.00 (Referent)	
BP-BES	44	6.1 (4.5-8.1)	1.19 (0.88-1.61)	0.26	1.11(0.80-1.54)	0.54
PtCr-EES	84	5.5 (4.4-6.8)	1.05 (0.82-1.35)	0.70	1.06 (0.82–1.38)	0.65
Re-ZES	53	4.7 (3.5-6.2)	1.13 (0.86-1.49)	0.37	1.11 (0.84-1.48)	0.45
Target vessel MI						
CoCr-EES	206	7.2 (6.4-8.3)	1.00 (Referent)		1.00 (Referent)	
BP-BES	36	4.7 (3.4-6.5)	0.62 (0.43-0.88)	0.007	0.77(0.53-1.12)	0.11
PtCr-EES	118	5.5 (4.5-6.3)	0.72 (0.58-0.91)	0.005	0.79(0.63-1.00)	0.05
Re-ZES	107	6.0 (5.0-7.3)	0.81 (0.64-1.02)	0.07	0.84(0.62-1.07)	0.15
Target vessel MI except periprocedural MI						
CoCr-EES	10	0.4 (0.2-0.4)	1.00 (Referent)		1.00 (Referent)	
BP-BES	6	0.9 (0.4-1.9)	1.48 (0.56-3.96)	0.43	1.43(0.50-4.10)	0.50
PtCr-EES	9	0.6 (0.3-1.1)	1.30 (0.57-2.94)	0.53	1.18(0.51-2.70)	0.70
Re-ZES	8	0.7 (0.4-1.5)	1.41(0.59-3.37)	0.43	1.24(0.51-3.05)	0.64
TVR						
CoCr-EES	132	6.0 (5.1-7.1)	1.00 (Referent)		1.00 (Referent)	
BP-BES	37	5.3 (3.9-7.3)	0.91 (0.66-1.27)	0.59	0.96(0.67-1.38)	0.83
PtCr-EES	91	6.1 (5.0-7.5)	1.00 (0.78-1.29)	0.98	1.02(0.79-1.32)	0.90
Re-ZES	82	6.8 (5.4-8.4)	1.12 (0.86-1.45)	0.42	1.17(0.89-1.53)	0.30

MI = myocardial infarction; TVR = target vessel revascularization; other abbreviations as in Table 1.

a 3-year event rates (%) determined by the Kaplan-Meier method.

b Target vessel failure was defined as death from cardiac causes, target vessel MI, or TVR.

**Figure 3:**
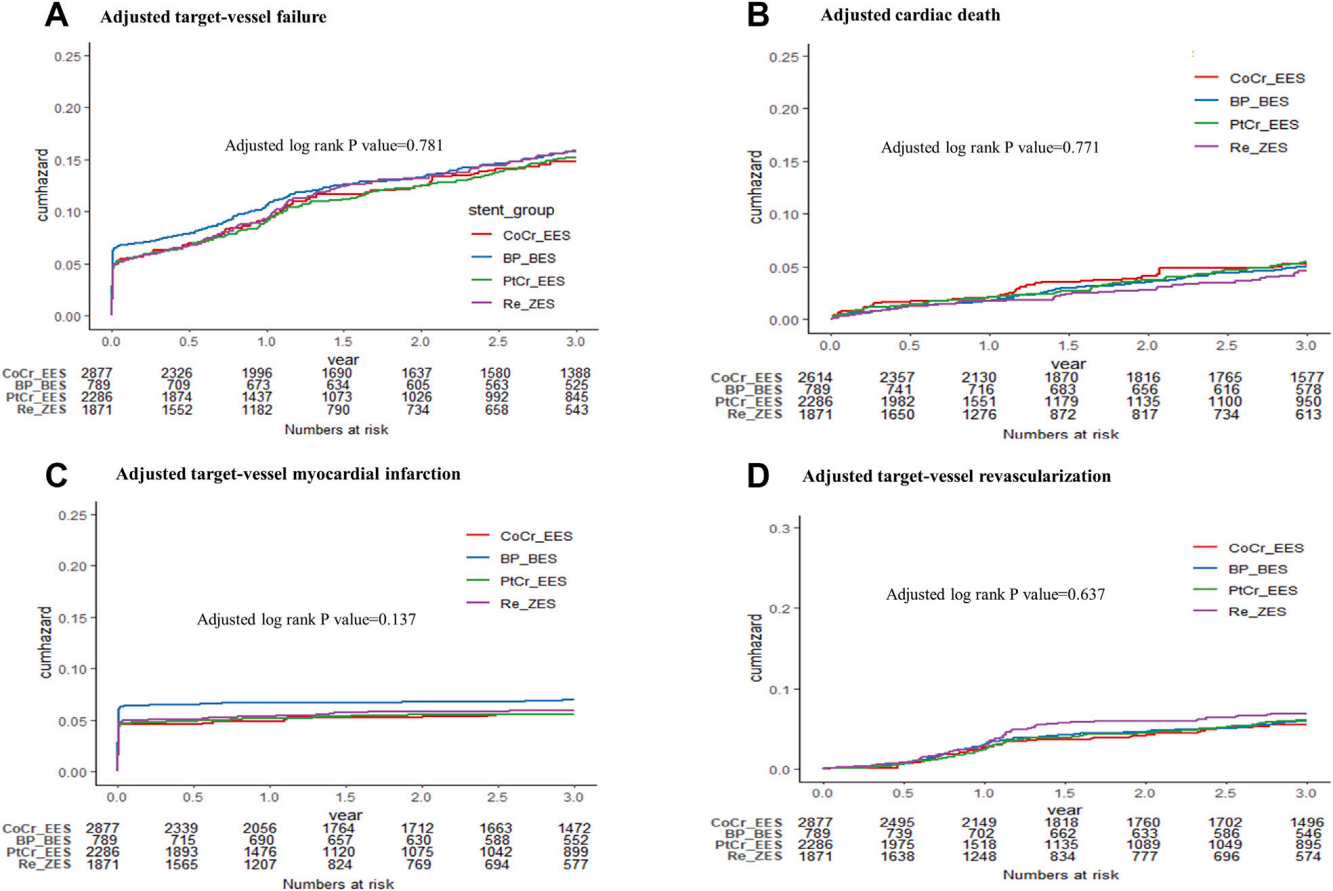
Adjusted Curves for the TVF and Its Components Adjusted cumulative-incidence curves for primary composite outcome of target vessel failure **(A)** and its individual components: cardiac death **(B)**, target vessel myocardial infarction **(C)**, and target vessel revascularization **(D**). *P* values were calculated using the adjusted log-rank test. Target vessel failure was defined as death from cardiac causes, target vessel myocardial infarction, or target vessel revascularization. Abbreviations as in Figure 1.

The adjusted risks for multiple DES comparisons after application of multiple treatment propensity-score weighting are shown in Table 2. Overall, there were no significant differences in adjusted risk for the primary composite outcome of TVR according to the different types of DES (adjusted log-rank *P* = 0.78) (Figure 3). There were also no between-group differences regarding the adjusted risks for individual components of TVF. With the CoCr-EES as the reference group, the adjusted HRs for TVF were similar for the BP-BES (HR: 0.96; 95% CI: 0.78-1.19; *P* = 0.72), PtCr-EES (HR: 0.94; 95% CI: 0.81-1.10; *P* = 0.43), and Re-ZES groups (HR: 1.02; 95% CI: 0.87-1.20; *P* = 0.79) (Central Illustration). This pattern was consistent for all pairwise comparisons of each DES (Supplemental Table 3).

**Central Illustration:**
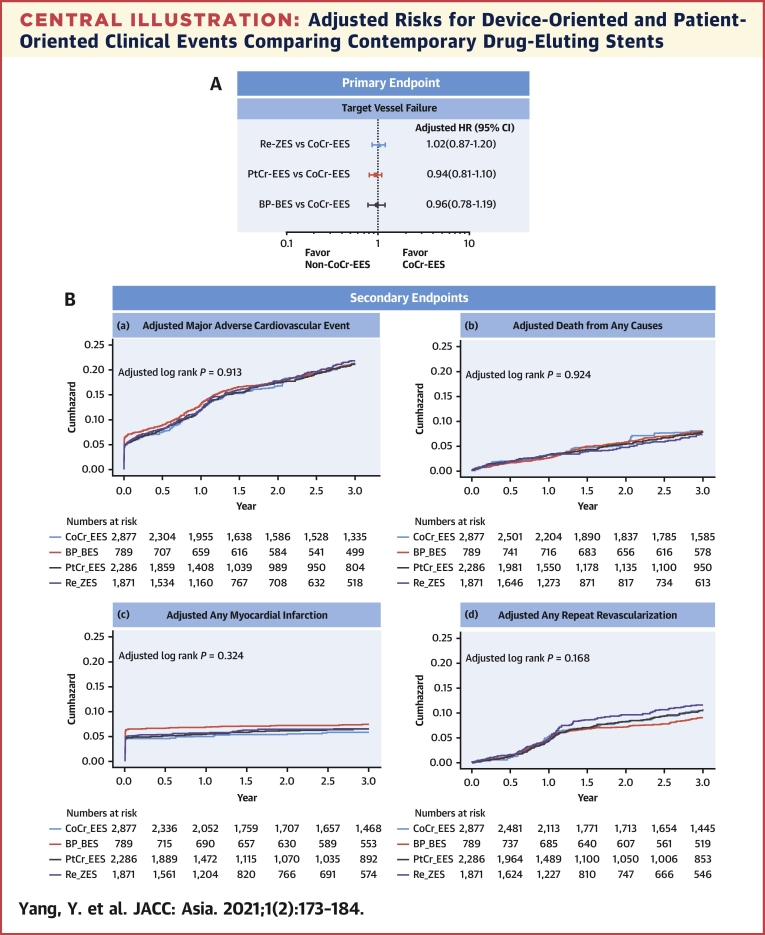
Adjusted Risks for Device-Oriented and Patient-Oriented Clinical Events Comparing Contemporary Drug-Eluting Stents **(A)** Adjusted HR using multigroup propensity-score analyses are given for different types of stent compared with the CoCr-EES. **(B)** Unadjusted Kaplan-Meier curves for cumulative incidence of secondary outcomes of **(a)** major adverse cardiac events, **(b)** all-cause mortality, **(c)** any myocardial infarction, and **(d)** any revascularization. BP-BES = biodegradable polymer biolimus-eluting stents; CoCr-EES = cobalt chromium everolimus-eluting stents; DM = diabetes mellitus; HR = hazard ratio; PtCr-EES = platinum chromium everolimus-eluting stents; Re-ZES = Resolute zotarolimus-eluting stents.

Crude and adjusted analyses of secondary outcomes are summarized in Table 3 and Supplemental Figures 2 and 3. Following multigroup propensity-score analyses, there were no significant between-group differences regarding the adjusted risks for MACE, all-cause mortality, MI, or revascularization. At 3 years, 24 cases (0.4%) of definite (n = 18) or probable (n = 6) stent thrombosis had occurred (12 for CoCr-EES, 9 for PtCr-EES, and 3 for Re-ZES), mostly within 30 days (n = 12) of the index procedure. No stent thrombosis was seen in the BP-BES group.

**Table 3:** Unadjusted and Adjusted Probabilities of the Secondary Outcomes

	Number of Events	3-Year Event Rate^a^ (% and 95% CI)	Unadjusted		Multigroup Propensity-Score Analysis	
			HR (95% CI)	*P* Value	HR (95% CI)	*P* Value
MACE^b^						
CoCr-EES	524	18.2 (16.8-19.6)	1.00 (Referent)		1.00 (Referent)	
BP-BES	153	19.4 (16.6-22.2)	0.96 (0.82-1.13)	0.64	1.02 (0.85-1.21)	0.86
PtCr-EES	360	15.8 (14.3-17.2)	0.95 (0.83-1.07)	0.40	0.98 (0.86–1.12)	0.79
Re-ZES	294	15.7 (14.1-17.4)	1.00 (0.88-1.15)	0.95	1.04 (0.90-1.19)	0.62
Death from any cause						
CoCr-EES	178	6.2 (5.3-7.1)	1.00 (Referent)		1.00 (Referent)	
BP-BES	61	7.7 (5.9-9.6)	1.06 (0.82-1.36)	0.68	1.04 (0.78-1.37)	0.80
PtCr-EES	125	5.5 (4.5-6.4)	1.01 (0.82-1.24)	0.92	1.01 (0.82-1.24)	0.94
Re-ZES	87	4.7 (3.7-5.6)	1.08 (0.86-1.34)	0.53	1.08 (0.86-1.36)	0.51
Any MI						
CoCr-EES	215	7.5 (6.5-8.4)	1.00 (Referent)		1.00 (Referent)	
BP-BES	39	4.9 (3.4-6.5)	0.64 (0.46-0.90)	0.009	0.78 (0.55-1.11)	0.17
PtCr-EES	129	5.6 (4.7-6.6)	0.79 (0.64-0.98)	0.03	0.86 (0.69-1.06)	0.16
Re-ZES	113	6.0 (5.0-7.1)	0.85 (0.68-1.06)	0.15	0.88 (0.70-1.11)	0.28
Any revascularization						
CoCr-EES	198	6.9 (6.0-7.8)	1.00 (Referent)		1.00 (Referent)	
BP-BES	73	9.3 (7.2-11.3)	1.21 (0.95-1.54)	0.12	1.22 (0.95-1.58)	0.12
PtCr-EES	153	6.7 (5.7-7.7)	1.09 (0.90-1.33)	0.39	1.11 (0.90-1.35)	0.33
Re-ZES	137	7.3 (6.1-8.5)	1.23 (1.00-1.51)	0.05	1.24 (1.01-1.53)	0.05
Stent thrombosis						
CoCr-EES	12	0.4 (0.2-0.7)	1.00 (Referent)		1.00 (Referent)	
BP-BES	0	0.00	NA		NA	
PtCr-EES	9	0.4 (0.1-0.7)	0.93 (0.40-2.17)	0.86	0.84 (0.35-1.97)	0.68
Re-ZES	3	0.2 (0.00-0.3)	0.53 (0.17-1.63)	0.27	0.42 (0.13-1.36)	0.15

MACE = major adverse cardiac events; NA = not available; other abbreviations as in Tables 1 and 2.

a 3-year event rates (%) determined by the Kaplan-Meier method.

b MACE was defined as the composite of all-cause death, any MI, and any repeat revascularization.

### Influence of insulin treatment

**Figure 4:**
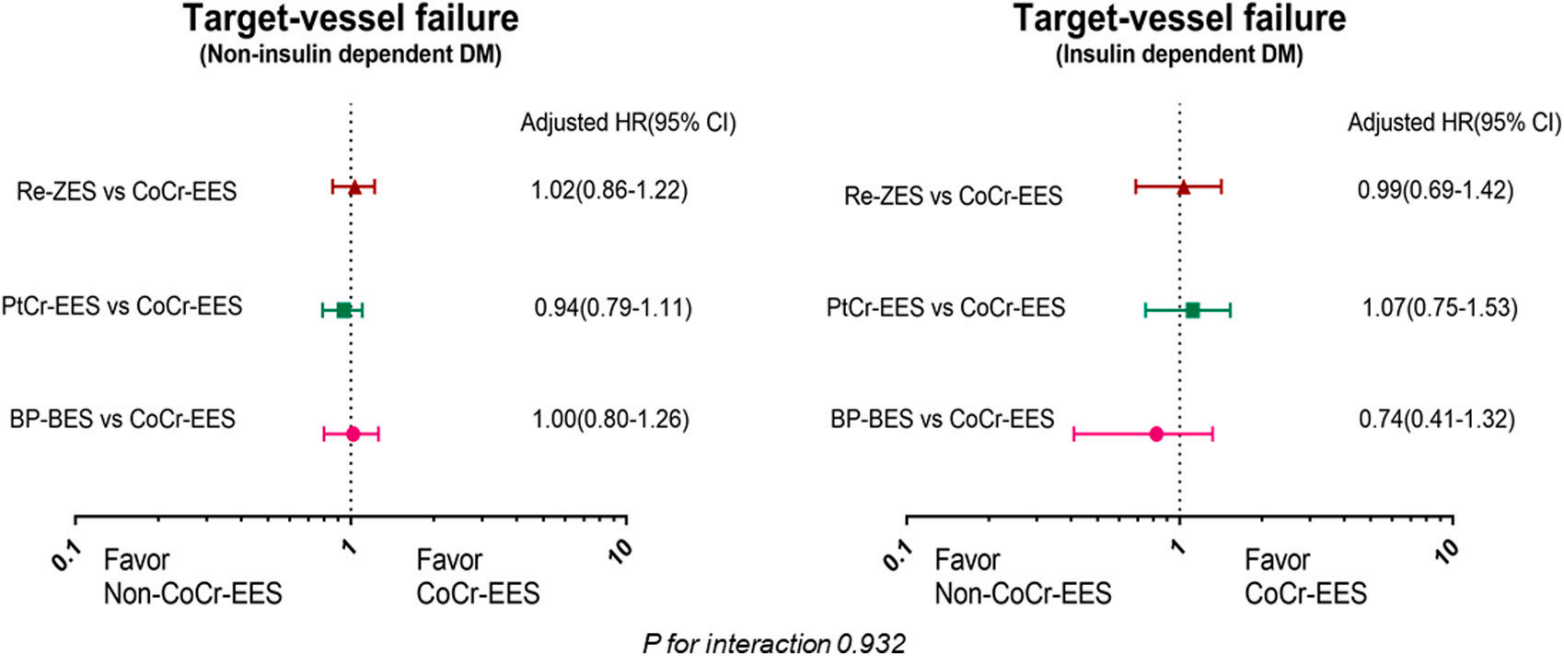
Adjusted HRs for TVF According to Insulin Use Adjusted HRs using multigroup propensity-score analyses are given for different types of stents compared with the CoCr-EES in in patients with non–insulin- or insulin-treated diabetes mellitus. Abbreviations as in Figure 1.

The rate of TVF was significantly higher in patients with insulin-treated DM than in those with non–insulin-treated DM (Supplemental Figure 4). The unadjusted and adjusted analyses of the primary composite outcome of TVF and its components at 3 years in patients with non–insulin-treated DM (n = 6,930) and those with insulin-treated DM (n = 893) are shown in Supplemental Tables 4 and 5, respectively. On multigroup propensity-score analyses, the adjusted risks of TVF were similar between different DES groups in patients with non–insulin-treated and insulin-treated DM (Figure 4). There was no significant interaction between stent group and insulin-dependency (*P*-for-interaction = 0.93).

### Influence of DM control

Among the total of 7,823 patients, only 4,989 (63.8%) had HbA_1c_ data at the index hospitalization. Among them, 2,292 patients (46.0%) had a HbA_1c_ level of ≤7 and 2,697 (54.0%) had a HbA_1C_ level >7. Baseline demographics, and clinical, anatomical, and procedural characteristics of patients with well-controlled DM (HbA_1c_ level of ≤7) or poor-controlled DM (HbA_1C_ level >7) according to the different types of DES are summarized in Supplemental Tables 6 and 7, respectively. Overall, there were significant between-group differences in sex, key clinical factors (dyslipidemia, prior history of PCI and coronary artery bypass graft, clinical presentation), several anatomical and procedural characteristics, and statin medication. The unadjusted and adjusted analyses of the primary composite of TVF and its components at 3 years in patients with HbA_1c_ ≤7 and those with HbA_1c_ >7 are shown in Supplemental Tables 8 and 9, respectively. There were no statistically significant differences in adjusted risk for TVF and its component according to the different types of DES in both groups of HbA_1c_ ≤7 and HbA_1c_ >7.

## Discussion

In this contemporary clinical-practice PCI registry study, we did not find significant differences between the adjusted risks of TVF and its components at 3 years across different types of second-generation contemporary DES in patients with DM. Moreover, there were no significant differences in the risks of patient-related outcomes of MACE and its components according to the types of DES. The incidence of stent thrombosis was considerably low (<1.0%) for all types of contemporary DES. The relative treatment effects for different types of DES were consistent in patients with non–insulin-treated DM and insulin-treated DM.

DM is a leading cause of obstructive coronary artery disease and may predispose to a more severe form of atherosclerotic coronary disease. As a result, DM has been regarded as a critical determinant for predicting poor prognosis and selecting optimal revascularization strategies among several clinical risk factors (11). With the marked advancements of PCI devices (from balloon angioplasty to bare-metal stents and first- and second-generation DES), technologies, experiences, and adjunctive drug therapies, PCI outcomes have dramatically improved over time even in patients with DM with complex coronary artery disease (12). Besides, DES have continuously undergone significant refinement and have become thinner, more deliverable, and more biocompatible. The combination of these factors has resulted in fewer local inflammatory reactions, less arterial injury, and reduced thrombogenicity (2). Likewise, given that PCI outcomes are rapidly improving and optimal medical therapy for DM is also continuously evolving (13), further comparative effectiveness researches are needed to determine the optimal choice of contemporary DES in high-risk patients with DM.

Some studies have compared second-generation DES with first-generation DES in patients with DM (4,14, 15, 16). Prior SPIRIT (Clinical Evaluation of the XIENCE V Everolimus Eluting Coronary Stent System) trial and subsequent patient-level meta-analysis comparing EES and paclitaxel-eluting stents (PES) (TAXUS, Boston Scientific) showed no significant differences in safety or efficacy between both types of stents in patients with DM (4,14). In a subgroup analysis of DM in the SCAAR (Swedish Coronary Angiography and Angioplasty Registry) comparing EES with first-generation sirolimus-eluting stents (SES) (Cypher, Cordis Corporation) and PES (TAXUS), the rates of death and stent thrombosis were significantly lower in the EES group compared with the SES and PES groups (15). The TUXEDO–India (Taxus Element versus Xience Prime in a Diabetic Population–India) trial showed that PES resulted in higher 1-year rates of TVF, MI, stent thrombosis, and TVR compared with EES (16). Although various second-generation DES have entirely replaced first-generation devices, each DES has a unique stent profile concerning the stent platform, polymer coating, and antiproliferative drug (17). However, there is a lack of data comparing the effectiveness and safety of second-generation DES in diabetic patients. Our large-sized, contemporary clinical practice registry involving unrestricted use of several second-generation DES showed the similar adjusted risks for TVF in pairwise comparisons of the different types of DES in patients with DM. The current findings might provide valuable clinical insights on the relative performance between different types of contemporary DES and help clinicians make the optimal choice of DES for diabetic patients in the real-world PCI setting.

A prior pooled analysis including 3 randomized trials (ISAR-TEST 3 [Rapamycin-Eluting Stents With Different Polymer Coating to Reduce Restenosis], ISAR-TEST 4 [Intracoronary Stenting and Angiographic Results: Test Efficacy of 3 Limus-Eluting Stents], and LEADERS [Limus Eluted From a Durable Versus Erodible Stent Coating]) showed similar 4-year rates of TVF between biodegradable polymer SES and durable polymer SES among diabetic subgroups. Interestingly, the rate of stent thrombosis was lower in BP-BES (18). In the 5-year follow-up of the COMPARE II (Comparison of the Everolimus Eluting With the Biolimus A9 Eluting Stent) trial comparing BP-BES and CoCr-EES in patients with DM, there were no differences for TVF and stent thrombosis (19), in which there was no case of stent thrombosis in the BP-BES group. Similarly, in our research, there was no case of stent thrombosis associated with the use of BP-BES. The BP-BES was created to reduce vessel wall inflammation caused by durable polymers and thus lower the occurrence of late stent thrombosis. However, given that overall rates of stent thrombosis for the various stent types were quite low, this finding should be interpreted as hypothesis-generating, and further largescale studies with longer-term follow-up are needed to compare the safety profiles of different contemporary DES in patients with DM.

As expected, an apparent gradient of the detrimental effect of DM according to treatment was observed in the current study; the 3-year rates of TVF were significantly higher in patients with insulin-treated DM than non–insulin-treated DM. Likewise, insulin-treated DM continues to be a strong risk factor for adverse outcomes in contemporary PCI practice with second-generation DES (20). In our study, regardless of the diabetic treatment, there were no between-group differences concerning measures of effectiveness, safety, and efficacy for the different types of DES. From a clinical viewpoint, insulin-treatment seems to influence the adverse clinical outcome but is not associated with the relative performance of each DES in diabetic patients. Further investigation is needed to determine whether DES performance may be influenced by either inherent differences between the insulin-deficient versus insulin-resistant state on the antirestenotic effects of eluting drugs or a direct inhibitory effect of insulin on the vascular response to specific drug analog-eluting stents (4).

### Study limitations

First, the study was observational; therefore, the overall findings should be considered hypothetical and hypothesis-generating. Second, the choice of the specific stents in our registries was not randomized, and thus, is subject to selection bias. Although we adjusted potentially confounding clinical variables, the comparative results could have been affected by unmeasured confounders. Third, our study was underpowered to detect clinically relevant differences of stent thrombosis rates between devices. Fourth, we categorized stent groups by the metallic platform and released drug, according to the manufacturer. Therefore, the stent group includes not just 1 specific stent model, but both previous and newer versions of stents. Finally, a longer-term follow-up is required to examine whether differences in late-occurring events between DES emerge over time; a final 5-year follow-up is currently being undertaken for each registry.

## Conclusions

In this contemporary clinical-practice registry study, there were no significant differences between the stent-related outcome of TVF and patient-related outcomes of MACE at 3 years among different types of second-generation DES in patients with DM undergoing PCI. These comparative outcomes were consistent in patients with non–insulin-treated and insulin-treated DM. Our findings should be confirmed or refuted through large, randomized clinical trials with long-term follow-up.

Perspectives **COMPETENCY IN MEDICAL KNOWLEDGE:** Limited data are available on the relative performance of different types of contemporary DES in patients with DM. Using a contemporary prospective clinical-practice registry involving unrestricted use of DES, we compared the relative effectiveness and safety profiles of various contemporary DES in patients with DM.

**COMPETENCY IN PATIENT CARE AND PROCEDURAL SKILLS:** On comparing 4 different types of DES (CoCr-EES, BP-BES, PtCr-EES, and Re-ZES), we found no significant between-group differences for risk of TVF in a 3-year follow-up of patients with DM.

**TRANSLATIONAL OUTLOOK:** Further research is needed to clarify the mechanisms underlying differences in vascular outcomes with various types of DES in patients with DM.

## Funding Support and Author Disclosures

This study was partly supported by the CardioVascular Research Foundation (Seoul, Republic of Korea). The sponsors had no role in the design and conduct of the study; collection, management, analysis, and interpretation of the data; preparation, review, or approval of the manuscript; or the decision to submit the manuscript for publication. The authors have reported that they have no relationships relevant to the contents of this paper to disclose.
