# Supplementary material for: Effectiveness and Safety of Contemporary Drug-Eluting Stents in Patients With Diabetes Mellitus
Source: JACC Asia. 2021 Sep 21;1(2):173–84. doi: 10.1016/j.jacasi.2021.07.009 (PMC9627859; doi:10.1016/j.jacasi.2021.07.009)
Supplement: Supplemental Figures 1–4 and Supplemental Tables 1–9 [file mmc1.docx]

**Supplemental Table 1. Baseline Characteristics of Patients with Non-Insulin-treated Diabetes Mellitus**

| **Variables** | **Non-insulin-treated Diabetes Mellitus (N = 6930)** | | | | |
| --- | --- | --- | --- | --- | --- |
|  | **CoCr-EES** | **BP-BES** | **PtCr-EES** | **Re-ZES** |  |
|  | **(N = 2496)** | **(N = 719)** | **(N = 2,051)** | **(N = 1,664)** | **p-value** |
| Age (years) | 65.4 ± 9.6 | 66.0 ± 10.2 | 66.1 ± 9.9 | 66.1 ± 9.9 | 0.03 |
| Male | 1717 (68.8) | 456 (63.4) | 1356 (66.1) | 1135 (68.21) | 0.02 |
| Body-mass index (kg/m^2^) | 24.9 ± 3.3 | 24.8 ± 3.2 | 24.8 ± 3.4 | 24.9 ± 3.3 | 0.72 |
| Hypertension | 1878 (75.2) | 525 (73.0) | 1522 (74.2) | 1288 (77.4) | 0.06 |
| Dyslipidemia | 1388 (55.6) | 251 (34.9) | 1189 (58.0) | 1011 (60.8) | <0.001 |
| Current smoking | 672(26.9) | 179 (24.1) | 473 (23.1) | 402 (24.2) | 0.02 |
| Family history of CAD | 131 (5.3) | 28 (3.9) | 94 (4.6) | 98 (5.9) | 0.13 |
| Previous MI | 143 (5.7) | 30 (4.2) | 153 (7.5) | 111 (6.7) | 0.008 |
| Previous heart failure | 71 (2.8) | 18 (2.5) | 75 (3.7) | 53 (3.2) | 0.32 |
| Previous PCI | 419 (16.8) | 70 (9.7) | 389 (19.0) | 290 (17.4) | <0.001 |
| Previous CABG | 62 (2.5) | 17 (2.4) | 35 (1.7) | 35 (2.1) | 0.33 |
| Chronic kidney disease | 163 (6.5) | 30 (4.2) | 132 (6.4) | 106 (6.4) | 0.12 |
| Previous cerebrovascular disease | 213 (8.5) | 67 (9.3) | 176 (8.6) | 137 (8.2) | 0.86 |
| Peripheral vascular disease | 59 (2.4) | 17 (2.4) | 55 (2.7) | 53 (3.2) | 0.41 |
| Chronic lung disease | 48 (1.9) | 19 (2.6) | 39 (1.9) | 39 (2.4) | 0.51 |
| Ejection fraction (%) | 57.9 ± 11.2 | 57.9 ±11.1 | 57.7 ± 10.9 | 57.5 ± 11.2 | 0.62 |
| Clinical presentation |  |  |  |  | <0.001 |
| Stable angina | 1225 (49.1) | 315 (43.8) | 833 (40.6) | 730 (43.9) |  |
| Unstable angina | 699 (28.0) | 214 (29.8) | 699 (34.1) | 543 (32.6) |  |
| NSTEMI | 320 (12.8) | 102 (14.2) | 319 (15.6) | 254 (15.3) |  |
| STEMI | 252 (10.1) | 88 (12.2) | 200 (9.8) | 137 (8.2) |  |
| Acute coronary syndrome | 1271 (50.9) | 404 (56.2) | 1218 (59.4) | 934 (56.1) | <0.001 |
| Number of diseased vessels | 1.85 ± 0.79 | 1.71 ± 0.77 | 1.81 ± 0.79 | 1.84 ± 0.79 | <0.001 |
| Multivessel disease | 1514 (60.7) | 369 (51.3) | 1178 (57.4) | 996 (59.9) | <0.001 |
| Moderate-to-severe calcification | 276 (11.1) | 47 (6.5) | 260 (12.7) | 177 (10.6) | <0.001 |
| Complex (ACC-AHA B2 or C type) lesion | 1994 (79.9) | 497 (69.1) | 1506 (73.4) | 1336 (80.3) | <0.001 |
| Bifurcation lesion | 875 (35.1) | 225 (31.3) | 460 (22.4) | 540 (32.5) | <0.001 |
| De novo lesion | 2361 (94.6) | 705 (98.1) | 1960 (95.6) | 1585 (95.3) | 0.002 |
| Diffuse lesion | 1490 (59.7) | 279 (38.8) | 1062 (51.8) | 957 (57.5) | <0.001 |
| Number of stents per patient | 1.77 ± 1.03 | 1.45 ± 0.75 | 1.63 ± 0.90 | 1.65 ± 0.93 | <0.001 |
| Total stent length per patient | 34.04 ± 19.48 | 25.47 ± 11.61 | 30.06 ± 15.51 | 32.07 ± 16.90 | <0.001 |
| Average stent diameter | 3.16 ± 0.43 | 3.10 ± 0.37 | 3.11 ± 0.44 | 3.12 ± 0.44 | <0.001 |
| IVUS | 1219 (48.8) | 208 (28.9) | 681 (33.2) | 772 (46.4) | <0.001 |
| Femoral approach | 1514 (60.7) | 398 (55.4) | 975 (47.5) | 869 (52.2) | <0.001 |
| Medication data |  |  |  |  |  |
| Statin | 2187 (87.6) | 613 (85.3) | 1864 (90.9) | 1534 (92.2) | <0.001 |
| Aspirin | 2463 (98.7) | 706 (98.2) | 2019 (98.4) | 1643 (98.7) | 0.68 |
| P2Y12 inhibitor | 2457 (98.4) | 704 (97.9) | 2022 (98.6) | 1645 (98.) | 0.35 |
| HbA1c at admission | 7.4 ± 1.4 | 7.4 ± 1.6 | 7.4 ± 1.4 | 7.3 ± 1.4 | 0.46 |

Results are reported as mean ± standard deviation or number (%)

Abbreviations: BP-BES, biodegradable polymer-biolimus-eluting stent; CoCr-EES, cobalt chromium everolimus-eluting stent; PtCr-EES, platinum chromium everolimus-eluting stent (Synergy stent); Re-ZES, resolute zotarolimus-eluting stent; CABG, coronary artery bypass graft; CAD, coronary artery disease; IVUS, intravascular ultrasound; MI, myocardial infarction; NSTEMI, non-ST-elevation myocardial infarction; PCI: percutaneous coronary intervention; STEMI, ST-elevation myocardial infarction

**Supplemental Table 2. Baseline Characteristics of Patients with Insulin-treated Diabetes Mellitus**

|  | **Insulin-treated Diabetes Meliitus (N = 893)** | | | | |
| --- | --- | --- | --- | --- | --- |
|  | **CoCr-EES** | **BP-BES** | **PtCr-EES** | **Re-ZES** |  |
| **Variables** | **(N = 381)** | **(N = 70)** | **(N = 235)** | **(N = 207)** | **p-value** |
| Age (years) | 65.1 ± 10.0 | 66.2 ± 9.2 | 66.3 ± 10.1 | 63.8 ± 9.7 | 0.06 |
| Male | 157 (41.2) | 26 (37.1) | 96 (40.9) | 70 (33.8) | 0.31 |
| Body-mass index (kg/m^2^) | 25.2 ± 3.5 | 24.5 ± 3.2 | 25.2 ± 3.2 | 25.1 ± 3.4 | 0.44 |
| Hypertension | 281 (73.8) | 54 (77.1) | 170 (72.3) | 152 (73.4) | 0.88 |
| Dyslipidemia | 199 (52.2) | 26 (37.1) | 140 (59.6) | 146 (7.5) | <0.001 |
| Current smoking | 91 (23.9) | 16 (22.9) | 41 (17.5) | 37 (17.9) | 0.17 |
| Family history of CAD | 13 (3.4) | 1 (1.4) | 6 (2.6) | 13 (6.3) | 0.12 |
| Previous MI | 30 (7.9) | 8 (11.4) | 20 (8.5) | 11 (5.3) | 0.35 |
| Previous heart failure | 12 (3.2) | 2 (2.9) | 7 (3.0) | 11 (5.3) | 0.50 |
| Previous PCI | 88 (23.1) | 9 (12.9) | 55 (23.4) | 48 (23.2) | 0.26 |
| Previous CABG | 12 (3.2) | 1 (1.4) | 5 (2.1) | 10 (4.8) | 0.33 |
| Chronic kidney disease | 87 (22.8) | 18 (25.7) | 54 (23.0) | 53 (25.6) | 0.85 |
| Previous cerebrovascular disease | 43 (11.3) | 7 (10.0) | 21 (8.9) | 21 (10.1) | 0.83 |
| Peripheral vascular disease | 22 (5.8) | 3 (4.3) | 19 (8.1) | 14 (6.8) | 0.60 |
| Chronic lung disease | 11 (2.9) | 3 (4.3) | 5 (2.1) | 5 (2.4) | 0.78 |
| Ejection fraction (%) | 56.6 ± 12.4 | 56.7 ± 12.0 | 57.7 ± 10.6 | 58.1 ±11.0 | 0.41 |
| Clinical presentation |  |  |  |  | 0.02 |
| Stable angina | 215 (56.4) | 30 (42.9) | 102 (43.4) | 111 (53.6) |  |
| Unstable angina | 104 (27.3) | 23 (32.9) | 79 (33.6) | 61 (29.5) |  |
| NSTEMI | 35 (9.2) | 14 (20.0) | 36 (15.3) | 19 (9.2) |  |
| STEMI | 27 (7.1) | 3 (4.3) | 18 (7.7) | 16 (7.7) |  |
| Acute coronary syndrome | 166 (43.6) | 40 (57.1) | 133 (56.6) | 96 (46.4) | 0.006 |
| No. of diseased vessels | 1.94 ±0.81 | 1.81 ± 0.75 | 1.92 ± 0.83 | 2.01 ± 0.79 | 0.34 |
| Multivessel disease | 243 (63.8) | 43 (61.4) | 145 (61.7) | 143 (69.1) | 0.38 |
| Moderate to severe calcification | 62 (16.3) | 8 (11.4) | 27 (11.5) | 19 (9.2) | 0.07 |
| Complex (ACC-AHA B2 or C type) lesion | 313 (82.2) | 57 (81.4) | 174 (74.0) | 168 (81.2) | 0.09 |
| Bifurcation lesion | 138 (36.2) | 21 (30.0) | 59 (25.1) | 73 (35.3) | 0.03 |
| De novo lesion | 356 (93.4) | 70 (100) | 227 (96.6) | 196 (94.7) | 0.07 |
| Diffuse lesion | 237 (62.2) | 28 (40.0) | 123 (52.3) | 120 (58.0) | 0.002 |
| No. of stents per patient | 1.86 ± 1.08 | 1.54 ± 0.97 | 1.71 ± 0.92 | 1.67 ± 0.91 | 0.03 |
| Total stent length per patient | 34.71 ± 19.19 | 27.26 ± 14.78 | 32.46 ± 18.30 | 33.75 ± 17.52 | 0.02 |
| Average stent diameter | 3.17 ± 0.43 | 3.02 ± 0.29 | 3.11 ± 0.43 | 3.17 ± 0.46 | 0.04 |
| IVUS | 186 (48.8) | 23 (32.9) | 88 (37.5) | 107 (51.7) | 0.001 |
| Femoral approach | 279 (73.2) | 48 (68.6) | 140 (59.6) | 128 (61.8) | 0.002 |
| Medication data |  |  |  |  |  |
| Statin | 331 (86.9) | 56 (80.0) | 205 (87.2) | 178 (86.0) | 0.45 |
| Aspirin | 374 (98.2) | 69 (98.6) | 227 (96.6) | 204 (98.6) | 0.46 |
| P2Y12 inhibitor | 374 (98.2) | 67 (95.7) | 230 (97.9) | 202 (97.6) | 0.65 |
| HbA1c at admission | 8.3 ± 1.7 | 8.0 ± 1.4 | 8.4 ± 1.6 | 8.1 ± 1.3 | 0.34 |

Results are reported as mean ± standard deviation or number (%).

Abbreviations are the same as in Online Table 1.

**Supplemental Table 3. Adjusted Hazard Ratios for Pairwise Comparison of the Primary Endpoint and Its Components by Multiple Treatment Propensity-Score Analysis**

|  | **Adjusted HR (95% CI)** | | | |
| --- | --- | --- | --- | --- |
| **Stent comparison** | **Target vessel failure** | **Cardiac death** | **Target vessel MI** | **TVR** |
| BP-BES vs. CoCr-EES | 0.96 (0.77–1.19) | 1.11 (0.80–1.54) | 0.77 (0.53–1.12) | 0.96 (0.67–1.38) |
| p-Value | 0.52 | 0.54 | 0.17 | 0.3 |
| PtCr-EES vs. CoCr-EES | 0.94 (0.81–1.10) | 1.06 (0.82–1.38) | 0.79 (0.63–1.00) | 1.02 (0.79–1.32) |
| p-Value | 0.43 | 0.65 | 0.046 | 0.90 |
| Re-ZES vs. CoCr-EES | 1.02(0.87-1.20) | 1.11 (0.841.48) | 0.84 (0.62–1.07) | 1.17 (0.89–1.53) |
| p-Value | 0.79 | 0.45 | 0.15 | 0.26 |
| PtCr-EES vs. BP-BES | 0.97 (0.78–1.22) | 0.96 (0.68–1.35) | 1.02 (0.73–1.63) | 1.06 (0.73–1.54) |
| p-Value | 0.84 | 0.81 | 0.91 | 0.77 |
| Re-ZES vs. BP-BES | 1.06(0.84–1.34) | 1.01 (0.70–1.44) | 1.09 (0.73–1.63) | 1.22 (0.83–1.79) |
| p-Value | 0.61 | 0.98 | 0.68 | 0.32 |
| PtCr-EES vs. Re-ZES | 0.92 (0.77–1.09) | 0.95 (0.71–1.29) | 0.94 (0.72–1.23) | 0.87 (0.65–1.16) |
| p-Value | 0.35 | 0.45 | 0.66 | 0.35 |

**Supplemental Table 4. Unadjusted and Adjusted Probabilities of the Primary Endpoint and Its Components in Patients with Non-Insulin-treated Diabetes Mellitus**

|  |  |  | **Unadjusted** | | **Multigroup Propensity-Score Analysis** | |
| --- | --- | --- | --- | --- | --- | --- |
| **Outcomes** | **No. of events** | **3-year event rate***  **(% and 95% CI)** | **HR (95% CI)** | **p-Value** | **HR (95% CI)** | **p-Value** |
| Target vessel failure† |  |  |  |  |  |  |
| CoCr-EES | 321 | 15.02 (13.54–16.64) | 1 (Referent) |  | 1 (Referent) |  |
| BP-BES | 91 | 13.52 (11.14–16.35) | 0.95 (0.77–1.17) | 0.63 | 1.00 (0.80–1.26) | 0.97 |
| PtCr-EES | 217 | 13.40 (11.75–15.25) | 0.90 (0.77–1.06) | 0.21 | 0.94 (0.79–1.11) | 0.44 |
| Re-ZES | 185 | 14.91 (12.89–1.21) | 1.00 (0.84–1.19) | 0.98 | 1.02 (0.86–1.22) | 0.82 |
| Cardiac death |  |  |  |  |  |  |
| CoCr-EES | 83 | 4.53 (3.65–5.61) | 1 (Referent) |  | 1 (Referent) |  |
| BP-BES | 37 | 5.58 (4.07–7.62) | 1.32 (0.95–1.84) | 0.09 | 1.18 (0.83–1.68) | 0.35 |
| PtCr-EES | 65 | 4.61 (3.59–5.89) | 1.06 (0.80–1.41) | 0.68 | 1.06 (0.79–1.43) | 0.69 |
| Re-ZES | 43 | 4.17 (3.04–5.72) | 1.15 (0.84–1.57) | 0.39 | 1.09 (0.79–1.50) | 0.61 |
| Target vessel MI |  |  |  |  |  |  |
| CoCr-EES | 163 | 6.66 (5.73–7.72) | 1 (Referent) |  | 1 (Referent) |  |
| BP-BES | 31 | 4.42 (3.13–6.24) | 0.63 (0.43–0.93) | 0.02 | 0.79 (0.52–1.19) | 0.26 |
| PtCr-EES | 101 | 5.12 (4.22–6.21) | 0.76 (0.60–0.98) | 0.03 | 0.82 (0.64–1.05) | 0.12 |
| Re-ZES | 92 | 5.98 (4.86–7.34) | 0.85 (0.66–1.09) | 0.20 | 0.87 (0.67–1.13) | 0.29 |
| TVR |  |  |  |  |  |  |
| CoCr-EES | 102 | 5.35 (4.42-6.47) | 1 (Referent) |  | 1 (Referent) |  |
| BP-BES | 29 | 4.63 (3.24–6.61) | 0.90 (0.62–1.31) | 0.58 | 0.91 (0.61–1.49) | 0.64 |
| PtCr-EES | 69 | 5.07 (3.99–6.42) | 0.96 (0.72–1.27) | 0.75 | 0.95 (0.71–1.28) | 0.76 |
| Re-ZES | 67 | 6.44 (5.04–8.23) | 1.12 (0.83–1.51) | 0.46 | 1.15 (0.85–1.57) | 0.36 |

*3-year event rates (%) determined by the Kaplan–Meier method.

†Target vessel failure was defined as death from cardiac causes, target vessel MI, or TVR.

**Supplemental Table 5. Unadjusted and Adjusted Probabilities of the Primary Endpoint and Its Components in Patients with Insulin-treated Diabetes Mellitus**

|  |  |  | **Unadjusted** | | **Propensity-Score Analysis** | |
| --- | --- | --- | --- | --- | --- | --- |
| **Outcomes** | **No. of events** | **3-year event rate***  **(% and 95% CI)** | **HR (95% CI)** | **p-Value** | **HR (95% CI)** | **p-Value** |
| Target vessel failure† |  |  |  |  |  |  |
| CoCr-EES | 83 | 24.71 (20.34–29.82) | 1 (Referent) |  | 1 (Referent) |  |
| BP-BES | 15 | 22.54 (14.22–34.65) | 0.74 (0.44–1.27) | 0.28 | 0.74 (0.41–1.32) | 0.31 |
| PtCr-EES | 46 | 29.30 (22.34–37.83) | 0.99 (0.70–1.40) | 0.97 | 1.07 (0.75–1.53) | 0.71 |
| Re-ZES | 58 | 22.46 (16.86–29.57) | 0.91 (0.67–1.24) | 0.54 | 0.99 (0.69–1.42) | 0.94 |
| Cardiac death |  |  |  |  |  |  |
| CoCr-EES | 27 | 9.18 (6.37–13.15) | 1 (Referent) |  | 1 (Referent) |  |
| BP-BES | 7 | 10.68 (5.24–21.13) | 0.85 (0.38–1.91) | 0.69 | 0.80 (0.32–1.99) | 0.62 |
| PtCr-EES | 19 | 13.81 (8.83–21.26) | 1.24 (0.72–2.14) | 0.44 | 1.22 (0.69–2.14) | 0.50 |
| Re-ZES | 17 | 9.77 (5.71–16.46) | 1.18 (0.69–1.99) | 0.55 | 1.13 (0.63–2.04) | 0.67 |
| Target vessel MI |  |  |  |  |  |  |
| CoCr-EES | 41 | 10.91 (8.14–14.54) | 1 (Referent) |  | 1 (Referent) |  |
| BP-BES | 5 | 7.42 (3.15–16.93) | 0.60 (0.24–1.52) | 0.28 | 0.73 (0.29–1.89) | 0.52 |
| PtCr-EES | 15 | 6.97 (4.16–11.55) | 0.61 (0.34–1.10) | 0.10 | 0.70 (0.38–1.29) | 0.25 |
| Re-ZES | 21 | 8.23 (5.35–12.55) | 0.63 (0.39–1.03) | 0.07 | 0.68 (0.37–1.23) | 0.20 |
| TVR |  |  |  |  |  |  |
| CoCr-EES | 30 | 10.47 (7.40–14.71) | 1 (Referent) |  | 1 (Referent) |  |
| BP-BES | 8 | 12.35 (6.35–23.27) | 1.20 (0.57–2.52) | 0.63 | 1.21 (0.54–2.69) | 0.64 |
| PtCr-EES | 22 | 16.54 (10.96–24.56) | 1.46 (0.86–2.48) | 0.16 | 1.53 (0.89–2.64) | 0.12 |
| Re-ZES | 23 | 8.43 (5.38–13.11) | 1.13 (0.67–1.91) | 0.64 | 1.27 (0.70–2.28) | 0.43 |

*3-year event rates (%) determined by the Kaplan–Meier method.

†Target vessel failure was defined as death from cardiac causes, target vessel MI, or TVR.

**Supplemental Table 6.** Baseline Characteristics of Patients with Well-Controlled Diabetes Mellitus (HbA1c level of ≤ 7) According to Different DES Type

|  | HbA1c ≤ 7 (N = 2292) | | | | |
| --- | --- | --- | --- | --- | --- |
|  | CoCr-EES | BP-BES | PtCr-EES | Re-ZES |  |
| Variables | (N = 770) | (N = 253) | (N = 714) | (N = 555) | p-value |
| Age (years) | 66.4 ± 9.6 | 66.9 ± 9.9 | 67.4 ± 9.5 | 67.3 ± 9.9 | 0.21 |
| Male | 528 (68.6) | 165 (65.2) | 444 (62.2) | 379 (68.3) | 0.04 |
| Body-mass index (kg/m^2^) | 25.0 ± 3.2 | 24.5 ± 3.2 | 24.7 ± 3.3 | 24.8 ± 3.3 | 0.10 |
| Hypertension | 598 (77.7) | 195 (77.1) | 554 (77.6) | 446 (80.4) | 0.57 |
| Dyslipidemia | 401 (52.1) | 82 (32.4) | 441 (61.8) | 326 (58.7) | <0.001 |
| Current smoking | 175 (22.7) | 60 (23.7) | 164 (23.0) | 100 (18.0) | 0.10 |
| Family history of CAD | 25 (3.2) | 12 (4.7) | 29 (4.1) | 33 (5.9) | 0.12 |
| Previous MI | 40 (5.2) | 10 (4.0) | 50 (7.0) | 39 (7.0) | 0.17 |
| Previous heart failure | 25 (3.2) | 9 (3.6) | 35 (4.9) | 16 (2.9) | 0.22 |
| Previous PCI | 119 (15.5) | 18 (7.1) | 145 (20.3) | 97 (17.5) | <0.001 |
| Previous CABG | 23 (3.0) | 3 (1.2) | 8 (1.1) | 6 (1.1) | 0.02 |
| Chronic kidney disease | 76 (9.9) | 18 (7.1) | 58 (8.1) | 39 (7.0) | 0.25 |
| Previous cerebrovascular disease | 73 (9.5) | 21 (8.3) | 67 (9.4) | 55 (9.9) | 0.91 |
| Peripheral vascular disease | 24 (3.1) | 6 (2.4) | 28 (3.9) | 23 (4.1) | 0.51 |
| Chronic lung disease | 23 (3.0) | 8 (3.2) | 21 (2.9) | 19 (3.4) | 0.96 |
| Ejection fraction (%) | 57.7 ± 11.2 | 59.4 ±10.6 | 57.9 ± 11.0 | 58.5 ±11.0 | 0.16 |
| Clinical presentation |  |  |  |  | 0.51 |
| Stable angina | 319 (41.4) | 113 (44.7) | 312 (43.7) | 250 (45.0) |  |
| Unstable angina | 255 (33.1) | 86 (34.0) | 221 (31.0) | 191 (34.4) |  |
| NSTEMI | 114 (14.8) | 35 (13.8) | 111 (15.5) | 71 (12.8) |  |
| STEMI | 82 (10.6) | 19 ( 7.5) | 70 ( 9.8) | 43 ( 7.7) |  |
| Acute coronary syndrome | 451 (58.6) | 140 (55.3) | 402 (56.3) | 305 (55.0) | 0.57 |
| No. of diseased vessels | 1.8 ± 0.8 | 1.7 ± 0.8 | 1.7 ± 0.8 | 1.8 ± 0.8 | 0.002 |
| Multivessel disease | 457 (59.4) | 122 (48.2) | 380 (53.2) | 331 (59.6) | 0.002 |
| Moderate to severe calcification | 82 (10.6) | 21 ( 8.3) | 102 (14.3) | 63 (11.4) | 0.04 |
| Complex (ACC-AHA B2 or C type) lesion | 594 (77.1) | 175 (69.2) | 532 (74.5) | 448 (80.7) | 0.002 |
| Bifurcation lesion | 164 (21.3) | 77 (30.4) | 109 (15.3) | 135 (24.3) | <0.001 |
| De novo lesion | 728 (94.5) | 248 (98.0) | 686 (96.1) | 524 (94.4) | 0.07 |
| Diffuse lesion | 428 (55.6) | 101 (39.9) | 372 (52.1) | 333 (60.0) | <0.001 |
| No. of stents per patient | 1.7 ± 1.0 | 1.4 ± 0.8 | 1.6 ± 0.8 | 1.7 ± 0.9 | 0.001 |
| Total stent length per patient | 30.9 ± 16.5 | 24.9 ± 11.5 | 29.3 ± 14.0 | 31.0 ± 15.5 | <0.001 |
| Average stent diameter | 3.1 ± 0.4 | 3.1 ± 0.4 | 3.1 ± 0.4 | 3.1 ± 0.4 | 0.32 |
| IVUS | 331 (43.0) | 64 (25.3) | 226 (31.7) | 250 (45.0) | <0.001 |
| Femoral approach | 447 (58.1) | 140 (55.3) | 306 (42.9) | 284 (51.2) | <0.001 |
| Medication data |  |  |  |  |  |
| Statin | 671 (87.1) | 216 (85.4) | 645 (90.3) | 512 (92.3) | 0.004 |
| Aspirin | 761 (98.8) | 249 (98.4) | 700 (98.0) | 548 (98.7) | 0.61 |
| P2Y12 inhibitor | 754 (97.9) | 247 (97.6) | 707 (99.0) | 548 (98.7) | 0.23 |

Results are reported as mean ± standard deviation or number (%).

Abbreviations are the same as in Online Table 1.

**Supplemental Table 7.** Baseline Characteristics of Patients with Poor-Controlled Diabetes Mellitus (HbA1C level >7) According to Different DES Type

|  | **HbA1c > 7 (N = 2697)** | | | | |
| --- | --- | --- | --- | --- | --- |
|  | **CoCr-EES** | **BP-BES** | **PtCr-EES** | **Re-ZES** |  |
| **Variables** | **(N = 956)** | **(N = 265)** | **(N = 864)** | **(N = 612)** | **p-value** |
| Age (years) | 64.0 ± 10.0 | 64.5 ± 9.8 | 65.0 ± 10.5 | 64.6 ± 10.2 | 0.25 |
| Male | 619 (64.7) | 157 (59.2) | 553 (64.0) | 388 (63.4%) | 0.43 |
| Body-mass index (kg/m^2^) | 24.8 ± 3.4 | 25.0 ± 3.3 | 24.9 ± 3.3 | 25.1 ± 3.5 | 0.53 |
| Hypertension | 697 (72.9) | 185 (69.8) | 615 (71.2) | 424 (69.3%) | 0.44 |
| Dyslipidemia | 493 (51.6) | 99 (37.4) | 516 (59.7) | 376 (61.4%) | <0.001 |
| Current smoking | 282 (29.5) | 70 (26.4) | 206 (23.8) | 166 (27.1%) | 0.06 |
| Family history of CAD | 48 (5.0) | 9 (3.4) | 37 (4.3) | 29 (4.7%) | 0.69 |
| Previous MI | 57 (6.0) | 15 (5.7) | 57 (6.6) | 35 (5.7%) | 0.89 |
| Previous heart failure | 26 (2.7) | 7 (2.6) | 30 (3.5) | 15 (2.5%) | 0.66 |
| Previous PCI | 143 (15.0) | 25 (9.4) | 153 (17.7) | 98 (16.0%) | 0.012 |
| Previous CABG | 17 (1.8) | 5 (1.9) | 13 (1.5) | 18 (2.9%) | 0.25 |
| Chronic kidney disease | 63 (6.6) | 14 (5.3) | 60 (6.9) | 39 (6.4%) | 0.81 |
| Previous cerebrovascular disease | 89 (9.3) | 29 (10.9) | 73 (8.4) | 36 (5.9%) | 0.04 |
| Peripheral vascular disease | 26 (2.7) | 4 (1.5) | 25 (2.9) | 13 (2.1%) | 0.55 |
| Chronic lung disease | 21 (2.2) | 6 (2.3) | 13 (1.5) | 9 (1.5%) | 0.58 |
| Ejection fraction (%) | 57.3 ± 11.6 | 57.0 ± 11.4 | 57.8 ±11.0 | 57.8 ± 11.3 | 0.63 |
| Clinical presentation |  |  |  |  | <0.001 |
| Stable angina | 449 (47.0) | 95 (35.8) | 300 (34.7) | 239 (39.1) |  |
| Unstable angina | 259 (27.1) | 92 (34.7) | 328 (38.0) | 211 (34.5) |  |
| NSTEMI | 124 (13.0) | 41 (15.5) | 146 (16.9) | 98 (16.0) |  |
| STEMI | 124 (13.0) | 37 (14.0) | 90 (10.4) | 64 (10.5) |  |
| Acute coronary syndrome | 507 (53.0) | 170 (64.2) | 564 (65.3) | 373 (60.9) | <0.001 |
| No. of diseased vessels | 1.8 ± 0.8 | 1.7 ± 0.8 | 1.8 ± 0.8 | 1.8 ± 0.8 | 0.19 |
| Multivessel disease | 570 (59.6) | 138 (52.1) | 515 (59.6) | 367 (60.0) | 0.12 |
| Moderate to severe calcification | 127 (13.3) | 15 (5.7) | 112 (13.0) | 66 (10.8) | 0.004 |
| Complex (ACC-AHA B2 or C type) lesion | 767 (80.2) | 199 (75.1) | 636 (73.6) | 495 (80.9) | 0.001 |
| Bifurcation lesion | 242 (25.3) | 55 (20.8) | 157 (18.2) | 135 (22.1) | 0.003 |
| De novo lesion | 907 (94.9) | 261 (98.5) | 829 (95.9) | 590 (96.4) | 0.06 |
| Diffuse lesion | 576 (60.3) | 107 (40.4) | 447 (51.7) | 357 (58.3) | <0.001 |
| No. of stents per patient | 1.7 ± 1.0 | 1.4 ± 0.8 | 1.7 ± 0.9 | 1.7 ± 0.9 | <0.001 |
| Total stent length per patient | 31.7 ± 16.5 | 24.9 ± 11.4 | 29.8 ± 15.0 | 31.9 ± 15.8 | <0.001 |
| Average stent diameter | 3.1 ± 0.4 | 3.0 ± 0.3 | 3.1 ± 0.4 | 3.1 ± 0.4 | 0.17 |
| IVUS | 393 (41.1) | 72 (27.2) | 266 (30.8) | 242 (39.5) | <0.001 |
| Femoral approach | 573 (59.9) | 147 (55.5) | 415 (48.0) | 295 (48.2) | <0.001 |
| Medication data |  |  |  |  |  |
| Statin | 841 (88.0) | 228 (86.0) | 800 (92.6) | 556 (90.8) | 0.001 |
| Aspirin | 941 (98.4) | 261 (98.5) | 851 (98.5) | 605 (98.9) | 0.91 |
| P2Y12 inhibitor | 942 (98.5) | 258 (97.4) | 850 (98.4) | 602 (98.4) | 0.62 |

Results are reported as mean ± standard deviation or number (%).

Abbreviations are the same as in Online Table 1.

**Supplemental Table 8.** Unadjusted and Adjusted Probabilities of the Primary End Point and Its Components in Patients with Well-Controlled Diabetes Mellitus (HbA1c level of ≤ 7) According to Different DES Type

|  |  |  | **Unadjusted** | | **Propensity-Score Analysis** | |
| --- | --- | --- | --- | --- | --- | --- |
| **Outcomes** | **No. of events** | **3-year event rate***  **(% and 95% CI)** | **HR (95% CI)** | **p-Value** | **HR (95% CI)** | **p-Value** |
| Target vessel failure† |  |  |  |  |  |  |
| CoCr-EES | 95 | 14.4 (11.7-17.1) | 1 (Referent) |  | 1 (Referent) |  |
| BP-BES | 28 | 12.0 (7.7-16.0) | 0.83 (0.56-1.22) | 0.34 | 0.80 (0.52-1.22) | 0.30 |
| PtCr-EES | 72 | 13.0 (10.0-16.0) | 0.96 (0.72-1.28) | 0.78 | 0.99 (0.74-1.33) | 0.96 |
| Re-ZES | 54 | 13.4 (9.7-16.9) | 0.99 (0.72-1.35) | 0.94 | 1.00 (0.73-1.38) | 0.99 |
| Cardiac death |  |  |  |  |  |  |
| CoCr-EES | 26 | 5.6 (2.9-6.4) | 1 (Referent) |  | 1 (Referent) |  |
| BP-BES | 13 | 5.6 (2.6-8.5) | 1.34 (0.76-2.36) | 0.31 | 1.07 (0.59-1.94) | 0.82 |
| PtCr-EES | 25 | 5.2 (3.1-7.3) | 1.21 (0.74-1.96) | 0.45 | 1.28 (0.78-2.10) | 0.34 |
| Re-ZES | 13 | 3.9 (1.6-6.1) | 1.20 (0.69-2.08) | 0.52 | 1.25 (0.71-2.19) | 0.45 |
| Target vessel MI |  |  |  |  |  |  |
| CoCr-EES | 44 | 5.9 (4.1-7.5) | 1 (Referent) |  | 1 (Referent) |  |
| BP-BES | 8 | 3.3 (1.0-5.5) | 0.54 (0.25-1.14) | 0.11 | 0.59 (0.25-1.40) | 0.23 |
| PtCr-EES | 35 | 5.2 (3.5-7.0) | 0.87 (0.56-1.36) | 0.54 | 0.90 (0.57-1.41) | 0.63 |
| Re-ZES | 26 | 5.2 (3.1-7.3) | 0.83 (0.51-1.36) | 0.47 | 0.83 (0.51-1.36) | 0.46 |
| TVR |  |  |  |  |  |  |
| CoCr-EES | 34 | 5.5 (3.7-7.3) | 1 (Referent) |  | 1 (Referent) |  |
| BP-BES | 9 | 4.1 (1.4-6.7) | 0.65 (0.32-1.31) | 0.23 | 0.72 (0.35-1.50) | 0.38 |
| PtCr-EES | 15 | 3.4 (1.7-5.2) | 0.66 (0.39-1.15) | 0.14 | 0.67 (0.38-1.16) | 0.15 |
| Re-ZES | 18 | 5.4 (2.8-7.9) | 0.96 (055-1.65) | 0.87 | 0.99 (0.56-1.73) | 0.97 |

*3-year event rates (%) determined by the Kaplan–Meier method.

†Target vessel failure was defined as death from cardiac causes, target vessel MI, or TVR.

**Supplemental Table 9.** Unadjusted and Adjusted Probabilities of the Primary End Point and Its Components in Patients with Poorly Controlled Diabetes Mellitus (HbA1C level >7) According to Different DES Type

|  |  |  | **Unadjusted** | | **Propensity-Score Analysis** | |
| --- | --- | --- | --- | --- | --- | --- |
| **Outcomes** | **No. of events** | **3-year event rate***  **(% and 95% CI)** | **HR (95% CI)** | **p-Value** | **HR (95% CI)** | **p-Value** |
| Target vessel failure† |  |  |  |  |  |  |
| CoCr-EES | 115 | 13.7 (11.3-16.0) | 1 (Referent) |  | 1 (Referent) |  |
| BP-BES | 38 | 15.3 (10.6-19.6) | 1.13 (0.81-1.59) | 0.48 | 1.38 (0.97-1.98) | 0.07 |
| PtCr-EES | 104 | 16.0 (13.0-19.0) | 1.10 (0.85-1.42) | 0.46 | 1.13 (0.87-1.46) | 0.37 |
| Re-ZES | 68 | 14.9 (11.3-18.3) | 1.07 (0.81-1.43) | 0.63 | 1.10 (0.82-1.48) | 0.51 |
| Cardiac death |  |  |  |  |  |  |
| CoCr-EES | 26 | 3.4 (2.1-4.7) | 1 (Referent) |  | 1 (Referent) |  |
| BP-BES | 15 | 6.1 (3.0-9.0) | 1.52 (0.87-2.66) | 0.15 | 1.82 (0.98-3.40) | 0.06 |
| PtCr-EES | 30 | 5.3 (3.3-7.2) | 1.30 (0.82-2.08) | 0.27 | 1.30 (0.81-2.08) | 0.29 |
| Re-ZES | 15 | 4.1 (1.9-6.3) | 1.34 (0.80-2.26) | 0.27 | 1.32 (0.77-2.26) | 0.31 |
| Target vessel MI |  |  |  |  |  |  |
| CoCr-EES | 57 | 6.1 (4.6-7.7) | 1 (Referent) |  | 1 (Referent) |  |
| BP-BES | 14 | 5.5 (2.6-8.2) | 0.86 (0.48-1.55) | 0.62 | 1.11 (0.60-2.04) | 0.74 |
| PtCr-EES | 42 | 5.2 (3.6-6.8) | 0.83 (0.55-1.23) | 0.35 | 0.90 (0.60-1.36) | 0.62 |
| Re-ZES | 28 | 4.8 (3.0-6.5) | 0.78 (0.50-1.23) | 0.28 | 0.82 (0.52-1.31) | 0.41 |
| TVR |  |  |  |  |  |  |
| CoCr-EES | 44 | 5.9 (4.2-7.6) | 1 (Referent) |  | 1 (Referent) |  |
| BP-BES | 16 | 6.8 (3.5-10.0) | 1.29 (0.77-2.18) | 0.33 | 1.39 (0.80-2.39) | 0.24 |
| PtCr-EES | 48 | 8.6 (6.1-10.9) | 1.49 (1.01-2.20) | 0.05 | 1.48 (1.00-2.20) | 0.05 |
| Re-ZES | 32 | 7.7 (5.0-10.3) | 1.29 (0.82-2.01) | 0.27 | 1.31 (0.83-2.07) | 0.24 |

*3-year event rates (%) determined by the Kaplan–Meier method.

†Target vessel failure was defined as death from cardiac causes, target vessel MI, or TVR

**Supplemental Figure 1. Plots Showing the Covariate Balance Before and After Weighting for Each Comparison**


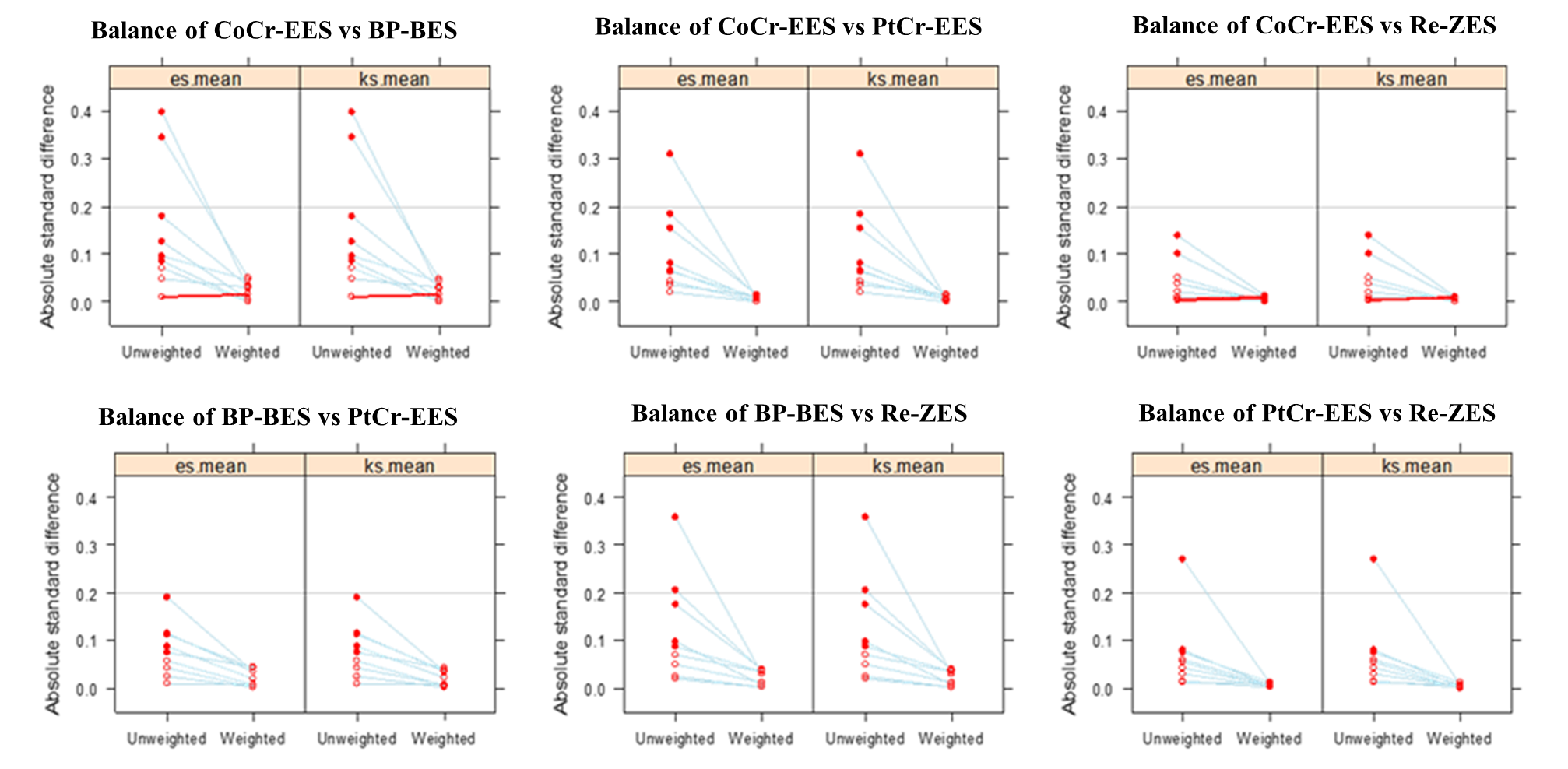


All values were less than 0.2. The solid circle indicates the statistically significant difference (before taking the maximum across treatment groups).

**Supplemental Figure 2. Unadjusted Kaplan-Meier Curves for Cumulative Incidence of Secondary Outcomes of (A) Major Adverse Cardiac Events, (B) All-Cause Mortality, (C) Any Myocardial infarction, and (D) Any Revascularization**


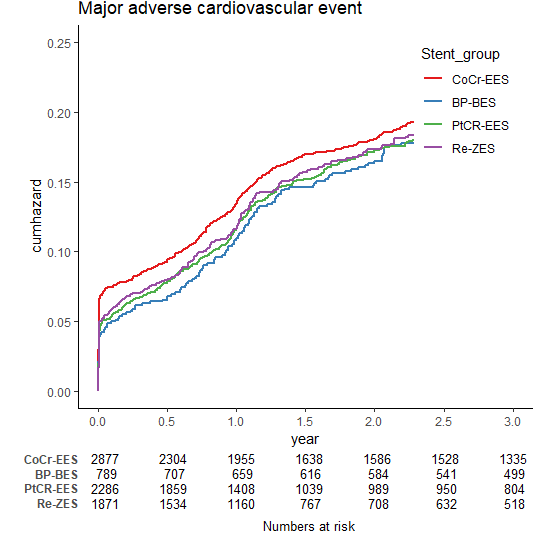


**(A) Major adverse cardiovascular event**

Log rank p value = 0.799


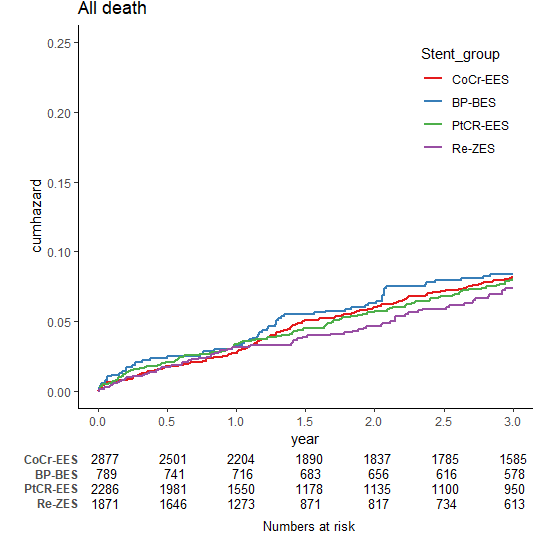


**(B) Death from any causes**

Log rank p value = 0.918


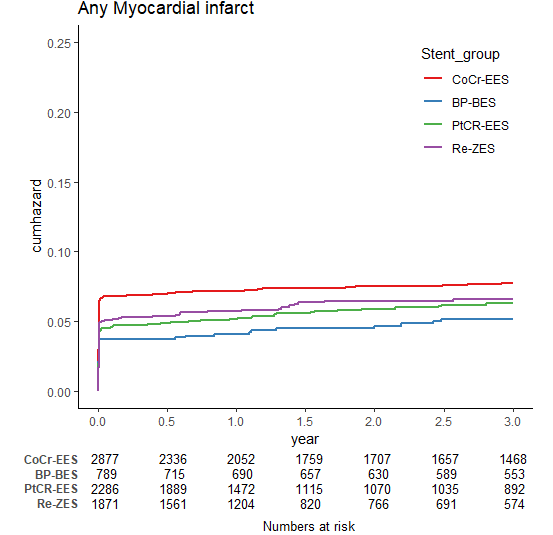


**(C) Any myocardial infarction**

Log rank p value = 0.028


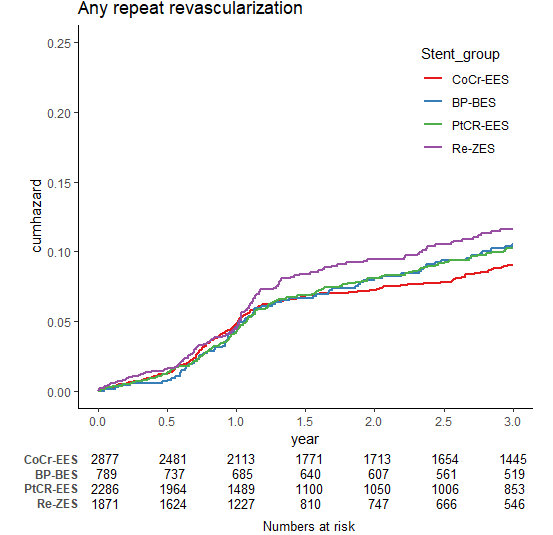


Log rank p value = 0.193

**(D) Any repeat revascularization**

**(C) All myocardial infarction**

**Supplemental Figure 3. Multigroup Propensity-Score Adjusted Kaplan-Meier Curves for Cumulative Incidence of Secondary Outcomes of (A) Major Adverse Cardiac Events, (B) All-Cause Mortality, (C) Any Myocardial infarction, and (D) Any Revascularizatio**

**
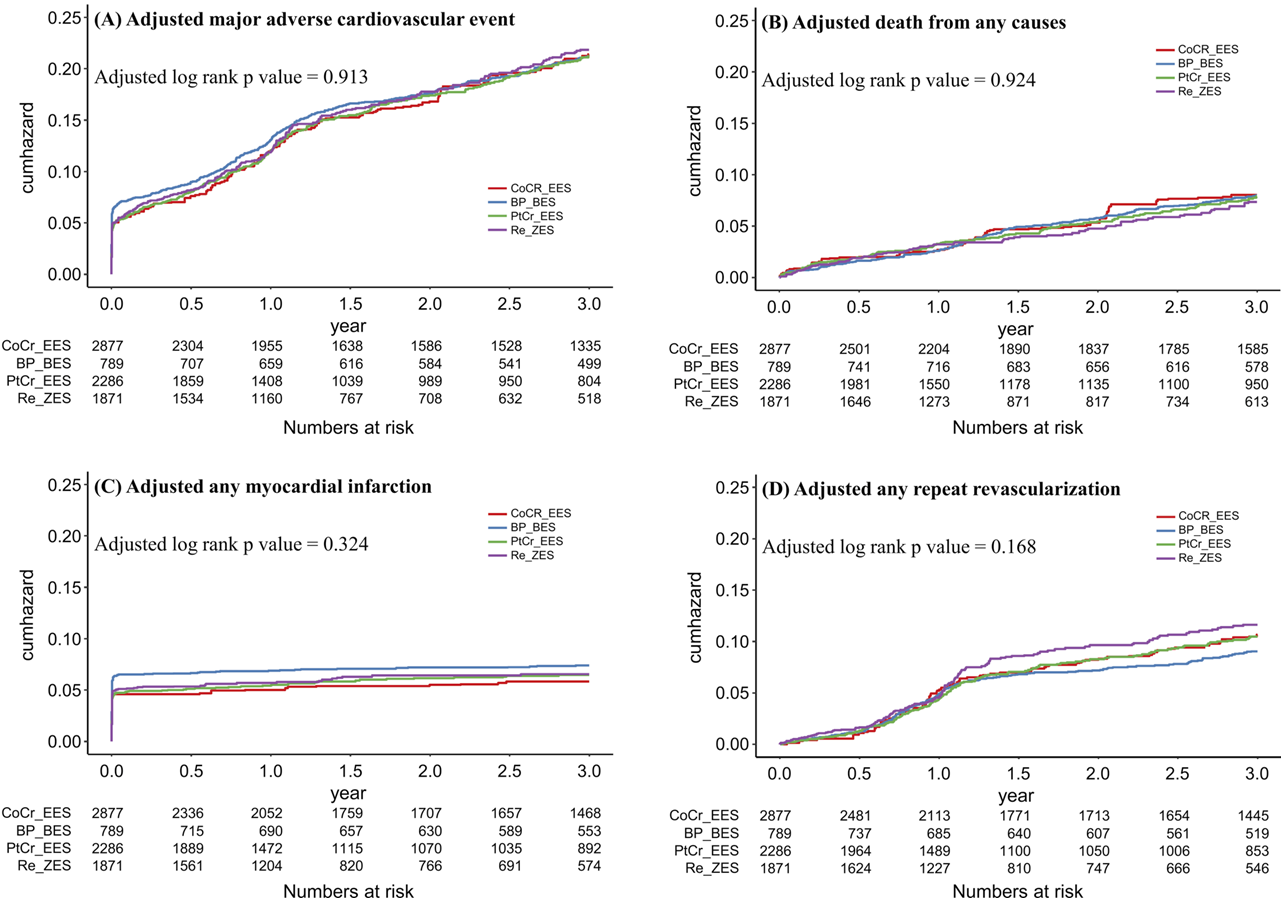
**

**Supplemental Figure 4. Unadjusted Kaplan–Meier Curves for Primary Composite Target Vessel Failure According to the Presence or Absence of Insulin Treatment**


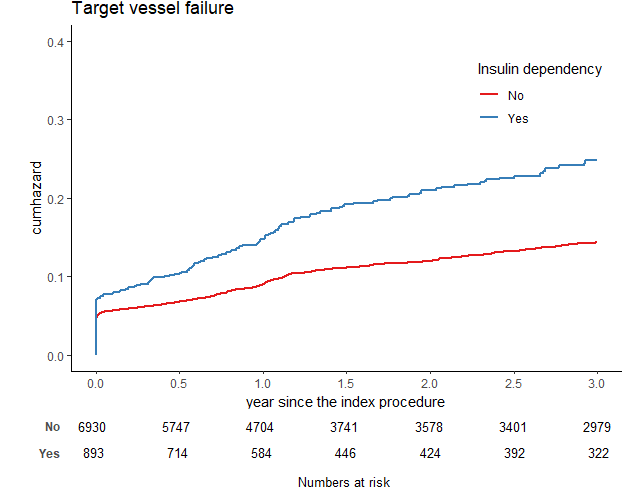


**24.8%**

**14.3%**

No

**Target vessel failure**

Yes

Insulin treated

Insulin treated

Yes

**P-value <0.001**
